# Infantile Hepatic Hemangioendothelioma in Comparison with Hepatoblastoma in Children: Clinical and Ultrasound Features

**DOI:** 10.5812/hepatmon.11103

**Published:** 2013-08-14

**Authors:** Fu-shun Pan, Ming Xu, Wei Wang, Lu-yao Zhou, Xiao-yan Xie

**Affiliations:** 1 Department of Medical Ultrasonic, The First Affiliated Hospital, Institute of Diagnostic and Interventional Ultrasound, Sun Yat-Sen University, Guangzhou, China

**Keywords:** Liver, Hemangioendothelioma, Hepatoblastoma, Ultrasonography

## Abstract

**Background:**

Infantile hepatic hemangioendothelioma (IHH) and hepatoblastoma (HBL) are respectively the most common benign and malignant liver tumors in children.

**Objectives:**

To study the clinical manifestations and the ultrasound features of the pediatric patients for distinguishing IHH from HBL.

**Patients and Methods:**

Between 2002 and 2012, thirteen children with IHH and 38 children with HBL under the age of 10 years were included. We retrospectively reviewed the clinical and the ultrasound features of the two groups, especially including parameters as follows: age at diagnosis, gender, alpha-fetoprotein (AFP) elevation, venous involvement and Doppler ultrasound.

**Results:**

Compared with HBL group, the age of IHH group was much younger (5.8 months vs. 35.1 months, P = 0.000), the AFP elevation was less likely to be detected in IHH group (23.1% vs. 89.5%, P = 0.000). Although the color flow were the same commonly observed (61.5% vs. 52.6%, P > 0.05), the spectral Doppler showed IHH was less likely to appear as arterial flow with resistance index (RI) > 0.7(12.5% vs. 75.0%, P < 0.05), characterized by arterial flow with RI < 0.7 and/or venous flow. Combined the clinical features including age (< 6 months) and normal AFP level yielded high capability in differential diagnosis, with sensitivity, specificity and Youden index of 77% (10/13), 95% (36/38), and 0.72, respectively. When combined clinical features (age and AFP) and spectral Doppler as the diagnostic criterion for distinguishing these cases with positive color flow signals, the sensitivity, specificity, accuracy and Youden Index were 88%, 95%, 89% and 0.83, respectively.

**Conclusions:**

The clinical features are effective indicators for distinguishing IHH from HBL, and the spectral Doppler may be a useful adjunct parameter for differential diagnosis.

## 1. Background

Liver tumors are rare in children, they account for about 5%-6% of all intra-abdominal masses in children, two-thirds of the pediatric primary liver masses are malignant (1). Infantile hepatic hemangioendothelioma (IHH) is the most common benign liver tumors in children with a peak presentation at 6 months of age. Unlike liver tumors in adults, in which the predominant malignant histology is hepatocellular carcinoma, hepatoblastoma (HBL) is the most common malignant pediatric liver tumors, and it is also the third of the most common fetal and neonatal liver tumors (1-3). Although they share some clinical manifestations, the treatment strategies and prognoses are quite different, the IHH may develop life-threatening complications including congestive heart failure and/or consumptive coagulopathy, it may regress spontaneously, therefore it needs a relatively conservative therapy especially when the infant is asymptomatic，while the HBL patients must undergo operation if possible because of its malignancy (4-6). Therefore the differential diagnosis is crucial to the clinicians for appropriate treatment selection.

The final diagnosis of pediatric liver tumors should be made in a stepwise approach and on the basis of the clinical features including age, gender and serum α-fetoprotein (AFP) level and imaging characteristics. Ultrasound is the preferred imaging method for evaluating children with liver tumors. It is not expensive but it can provide real-time assessment,, without ionizing radiation.

## 2. Objectives

To our knowledge, few studies were performed to assess the differential diagnosis value of the combined use of the clinical manifestations and the ultrasound features. The aim of this study was to evaluate the clinical manifestations combined with the ultrasound features of the children in differentiating IHH from HBL.

## 3. Patients and Methods

### 3.1. Patients

From March 2002 to July 2012, we retrospectively analyzed 51 consecutive children with HBL or IHH under the age of 10 years who had undergone abdominal ultrasound scanning before treatments in our institution. They were classified into the HBL group (n = 38) and the IHH group (n =13). The HBL group included 23 boys and 15 girls ranging in age from 1 month to 10 years. The IHH group consisted of 7 boys and 6 girls ranging in age from 1 day to 5 years. Among the 51 children, 45 (36 with HBL, 9 with IHH) underwent contrast enhanced CT, only 11 (6 with HBL, other 5 with IHH) underwent contrast MRI.

The IHH group obtained pathological diagnosis after surgery in 9 and clinical diagnosis in 4 on the basis of typical contrast enhanced CT imaging findings and the demonstration of involution at follow-up. Nine patients underwent partial hepatectomy and got pathological diagnosis, two patients received corticosteroids and/or interferon-alpha, whereas the remaining two patients were observed without any treatment. All the HBL patients obtained pathological diagnosis which was conﬁrmed by liver biopsy histopathology examination of specimens obtained from surgery. Among the 38 patients, 35 underwent partial hepatectomy and 3 received liver transplantation. Chemotherapy was given to all HBL patients. In the HBL group, 22 and 16 patients respectively got pathological diagnosis as epithelial type and mixed epithelial/mesenchymal type, including one small cell undifferentiated type. Written informed consent was obtained from all patients’ parents, and the study was approved by the Ethical Committee of the institution.

### 3.2. Ultrasound Examination and Imaging Analysis

Three US machines were used in this study depending on the availability. They were HDI 5000 (ATL/Philips, Bothell, WA), Acuson Sequoia 512 (Siemens Medical Solutions, Mountain View, CA) and GE Logiq 500 (GE Medical Systems, Waukesha, WI, USA). Convex transducers with frequency range from 6 MHz to 8 MHz and linear transducers with frequency range from 8 MHz to15 MHz were used. All the patients were examined by a radiologist who had more than 5 years’ experience in pediatric ultrasound. All patients had been fed nothing for at least 4 hours before the ultrasound examination. The ultrasound examination was done in supine position. The ultrasound features of the lesions including number, size, location, calcification within the mass together with the color Doppler flow imaging, and the conditions of the portal or hepatic venous involvement were evaluated and recorded.

The ultrasound images were independently analyzed by two staff radiologists, who had at least 10 years’ experience in pediatric ultrasound. Both specialists were blinded to the clinical history. Other imaging and pathological results were reviewed and the images and recorded ultrasound features for the same contents were randomly reviewed as mentioned above. We recorded the lesion number as solitary or multiple. The lesion size was recorded as the long axis diameter of the mass or the maximum lesion size in multiple lesions cases. The lesion location was recorded as the left, right or bi-lobe of the liver. Color and spectral Doppler of the lesion were evaluated, including color flow pattern (arterial and/or venous flow), flow velocity and resistant index (RI). Venous involvements including portal vein, hepatic vein and inferior vena cave were also recorded.

### 3.3. Statistical Analysis

All data analyses were performed with the SPSS statistical package (ver. 13.0 for Windows, SPSS Inc, Chicago, IL, USA). All values of continuous variables were expressed as mean ± standard error (SE). The differences in the clinical and ultrasound features between the two groups were assessed by Student’s *t*-test, Wilcoxon’s rank sum test or Chi-Square test. Statistical significance was defined as* P *< 0.05. Sensitivity, speciﬁcity, accuracy, positive predictive value (PPV), negative predictive value (NPV) and Youden Index were also calculated. The Youden index is a summary measure of accuracy incorporating both sensitivity and specificity, which was calculated as (sensitivity + specificity - 1). Whether the AFP level elevated or not, patients less than two years old were referred to the normal reference range given by Blohm et al(7).

## 4. Results

### 4.1. Clinical Presentation

The demographic and clinical characteristics of the IHH group and HBL group were listed in Table 1. There was no significant difference in the variables including gender distribution, cutaneous hemangioma and congestive heart failure (CHF) ( *P *>0.05). The palpable abdominal mass was more likely to be detected in HBL group than IHH group, 73.7% vs. 30.8% ( *P *=0.009). The AFP elevation was found less frequently in IHH group than HBL group, 23.1% vs. 89.5% ( *P *<0.001). The mean AFP levels of the IHH and HBL group were 3,625 ng/ml and 123,560 ng/ml respectively ( *P *<0.001). The age at diagnosis was younger than 6 months in 11(84.6%) cases of the IHH group, whereas older than 6 months in 32 (84.2%) of the HBL group. That is to say, the average age at diagnosis was younger in the IHH group in comparison with the HBL group. 

**Table 1.** Demographic and Clinical Characteristic of IHH and HBL

Characteristics	IHH^a^ (n = 13)	HBL^a^(n = 38)	*P value*
**Male, No. (%)**	7 (53.8)	23 (60.5)	0.750
**Palpable abdominal mass, No. (%)**	4 (30.8)	28 (73.7)	0.009
**Cutaneous hemangioma, No. (%)**	2 (15.4)	0 (0)	0.061
**CHF , No. (%)** ^**a**^ **, No. (%)**	2 (15.4)	0 (0)	0.061
**AFP elevation, No. (%)**	3 (23.1)	34 (89.5)	0.000
**AFP level (ng/ml), No. (%)**	3.625 ± 5.673	123.560 ± 157.812	0.000
**Age at diagnosis (mo), No. (%)**	5.8 ± 7.8	35.1 ± 20.3	0.000

^a^ Abbreviations: CHF, congestive heart failure; mo, months; IHH, infantile hepatic hemangioendothelioma; HBL, hepatoblatoma

### 4.2. Ultrasound Characteristics

**Figure 1.**
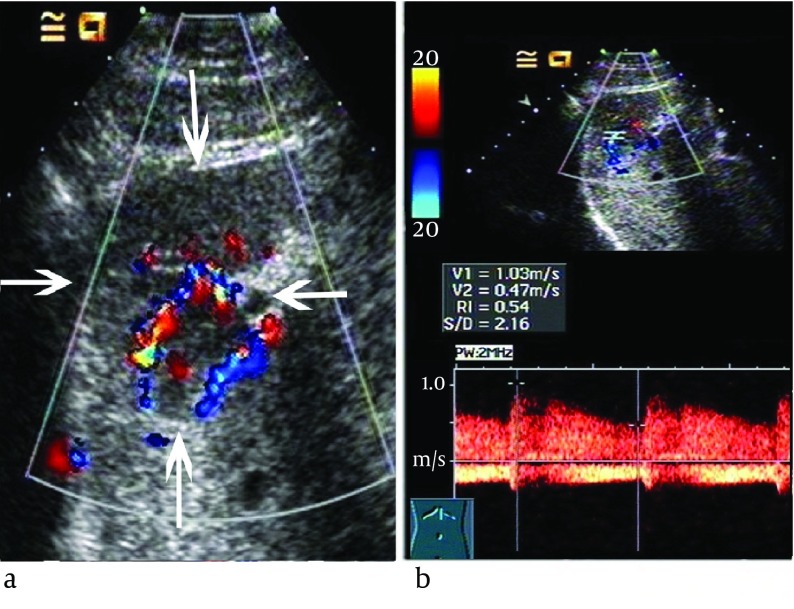
Infantile Hepatic Hemangioendothelioma in a 5-Month-Old Girl (a) Color Doppler revealed plenty of color flow within and adjacent to the nodule (arrows), 2.7 cm in size, in segments 2. (b) Spectral Doppler showed arterial flow with PSV of 103 cm/s and RI of 0.54

**Figure 2.**
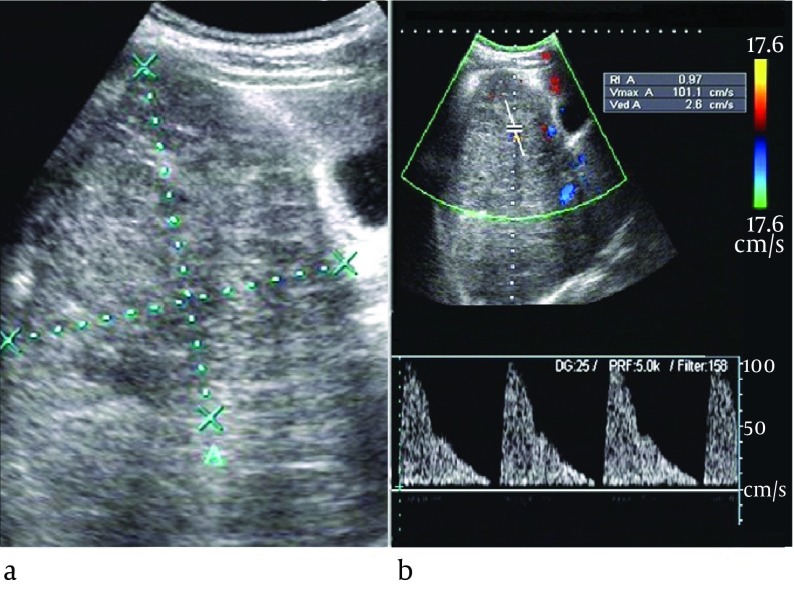
Hepatoblastoma in a 1-Year-Old Girl (a) Gray-scale US image showed an isoechoic nodule (calipers) 5.8 cm in size, in segment 5 of the liver. (b) Spectral Doppler showed arterial flow with PSV of 101.1 cm/s and RI of 0.97

The ultrasound features of the two groups were listed in Table 2. There was no significant difference in the location, the number of the tumors or calcification within the lesion between the two groups (P > 0.05). Although there was no patient with vein tumor thrombus in IHH group; the difference of vein involvement incidence was not significant in comparison with HBL group (P > 0.05). The mean diameter was much smaller in IHH group than HBL group (P < 0.05), (4.5 ± 1.3) cm and (7.1 ± 1.9) cm respectively. Color Doppler showed that color flow was occasionally detected in IHH and HBL group without significant difference, 61.5% vs. 52.6% (P > 0.05). Among the 8 color Doppler positive IHH patients, the spectral Doppler showed variable flow patterns: venous flow only in one case, arterial flow and/or venous flow in seven cases (Figure 1) and RI > 0.7 appeared only in one case. Between the 20 HBL patients with positive color Doppler arterial flow, 15 cases had RI > 0.7 (Figure 2). The spectral Doppler revealed a significant difference (P = 0.004), that is, the HBL group was more prone to appear as arterial flow with RI > 0.7. 

**Table 2.** Ultrasound Features of IHH and HBL

Characteristics	IHH (n = 13)	HBL (n = 38)	*P value*
**Lesion location, No. (%)**			0.668
**Lt^a^**	30.7 (4/13)	39.5 (15/38)	
**Rt^a^**	46.2 (6/13)	47.3 (18/38)	
**Bi^a^**	23.1 (3/13	13.2 (5/38)	
**Lesion number, No. (%)**			0.208
**Solitary**	69.2 (9/13)	86.8 (33/38)	
**Multiple**	30.8 (4/13)	13.2 (5/38)	
**Lesion size, cm**	4.5 ± 1.3	7.1 ± 1.9	0.000
**Vein tumor thrombus , No. (%)** ^**b**^ ****	0 (0/13)	18.4 (7/38)	0.230
**Lesion Calcification, No. (%)**	38.5 (5/13)	57.9 (22/38)	0.336
**positive flow signals **	61.5 (8/13)	52.6 (20/38)	0.749
**Spectral Doppler**			0.004
**RI >0.7**	12.5 (1/8)	75.0 (15/20)	
********RI< 0.7 **and/or** venou**s flow**	87.5 (7/8)	25.0 (5/20)	

^a^ Abbreviations: Lt, left; Rt, right; Bi, bi-lobe

^b^ refers to venous involvement including portal vein, hepatic vein and inferior vena cave; RI: resistance index

### 4.3. Diagnosis and Differential Diagnosis

The diagnosis value was analyzed on the basis of clinical presentation and ultrasound features (Table 3). When coupled age (< 6 months) and normal AFP level, the diagnosis value was as follows: sensitivity of 77% (10/13), specificity of 95% (36/38), accuracy of 90% (46/51), positive predictive value (PPV) of 83% (10/12) and negative predictive value (NPV) of 92% (36/39). When used spectral Doppler characterized by arterial flow with RI <0.7 and/or venous flow as indicator for distinguishing these cases with positive color flow signals, the sensitivity, specificity, accuracy and Youden Index were 88%, 75%, 79% and 0.63, respectively. When combined these indicators mentioned above for distinguishing these cases with positive color flow signals, the sensitivity, specificity, accuracy and Youden Index were 88%, 95%, 89% and 0.83, respectively. 

**Table 3.** Diagnostic Value of Clinical and Ultrasound Indicators for IHH

Indicators (clinical and US)	Sensitivity, No. (%)	Specificity, No. (%)	Accuracy, No. (%)	PPV^a^, No. (%)	NPV^a^, No. (%)	Youden Index
**Age < 6 month (1)**	85 (11/13) ^b^	84 (32/38)	84 (43/51)	65 (11/17)	94 (32/34)	0.69
**Normal AFP (2)**	77 (10/13)	89 (34/38)	86 (44/51)	71 (10/14)	92 (34/37)	0.66
**(1) + (2)**	77 (10/13)	95 (36/38)	90 (46/51)	83 (10/12)	92 (36/39)	0.72
**(3)**	88 (7/8)	75 (15/20)	79 (22/28)	58 (7/12)	94 (15/16)	0.63
**(1)+(2)+(3) ** ^**c**^	88 (7/8)	95 (19/20)	93 (26/28)	88 (7/8)	95 (19/20)	0.83

^a^ Abbreviations: PPV, positive predictive value; NPV, negative predictive value

^b^ Numbers in parenthesis: case number

^c^ (3) refers to spectral Doppler characterized by arterial flow with RI < 0.7 and/or venous flow

## 5. Discussion

IHH is the most common benign tumor of the liver in children, which accounts for 12% of all pediatric liver tumors and HBL is the most common malignant liver tumor in children, which accounts for 40-60% of all pediatric liver tumors (1-6). Histopathologically, IHH is recognized as a mesenchymal tumor composed of thin vascular channels lined by a single layer of plump endothelial cells within a scanty fibrous stroma (8). Most IHH cases lack symptoms and regress spontaneously without treatment(9), thus the actual incidence of IHH may be higher than 12% of all pediatric liver tumors. The treatment algorithm bases on the imaging features of the lesions and the presence or absence of complications(10). HBL is classified by histopathology as epithelial type or mixed epithelial/mesenchymal type. Surgical resection is the mainstay of treatment for HBL, and with the use of neoadjuvant chemotherapy, up to 85% of HBL cases become resectable and can be cured(11).

The age of diagnosis of IHH and HBL is notable, nearly 86% of IHH cases are diagnosed in the first 6 months of life, and approximately 30-50% of the HBL cases occurre in the first year of childhood (1-3). The IHH is also the most common fetal and neonatal liver tumors(2); which may develop apparent or severe clinical complications such as palpable abdominal mass, cutaneous hemangioma and congestive heart failure (6, 10). In our series, the mean age of diagnosis of IHH and HBL was 5.8 months and 35.1 months respectively. Three IHH cases and thirty-two HBL cases were older than 6 months, all asymptomatic, and we speculated that lack of symptoms might result in their delayed diagnosis. Additionally, although the palpable abdominal mass was more likely to be detected in HBL group than IHH group, from birth to a palpable abdominal mass may be a long time, which might be the reason for the older mean age of diagnosis of HBL than IHH.

Serum AFP is a useful laboratory marker for the differential diagnosis of pediatric liver tumors. AFP concentrations are normally elevated at birth, up to 40,000 ng/mL, decrease rapidly after birth, and do not reach the normal adult level until 6 months of age(7). AFP levels are rarely elevated above the normal reference range for age in IHH patients, while nearly 90% of HBL tumors consist of hepatoblast-like cells which secrete large amounts of AFP (2, 3, 5, 11). Unambiguous etiology of increased serum AFP in IHH is not well-established, recent study has shown that hepatocytes near or entrapped within the IHH tumor might be the source of the increased serum levels(12).

In our study, there were some similar clinical features between IHH and HBL. Firstly, similar to the HBL group, the IHH group also showed a male predominance, which was contrary to previous studies (3, 4, 6, 10). Small sample of our IHH group might be the reason. Secondly, although there was no HBL patient suffering from cutaneous hemangioma and CHF, only four in IHH group suffered from these two complications and no statistical difference was detected (*P* > 0.05). The precise incidences of cutaneous hemangioma and CHF in patients with IHH are unclear because of the wide disparity in different studies (3,10,13). Thus, the two clinical features should not be considered as an effective indicator for distinguishing IHH from HBL.

The imaging findings play a vital role for the diagnosis and treatment strategies selection forpediatric liver tumors. There are several modalities in common use including CT, MRI and ultrasound. With the improvements of these imaging techniques, findings of dynamic contrast-enhanced CT and MR are often specific and diagnostic for IHH (4, 14); however, it is deemed inappropriate to take CT or MR as the first-line imaging method for children with suspected liver mass due to its radiation or high cost. Ultrasound can provide real-time assessment, it is not expensive, without ionizing radiation, and moreover, it helps to evaluate the hepatic and portal venous involvement (15).

In nearly 50% cases of the both groups, the lesion was located in the right lobe of the liver, which was consistent with previous studies (1, 3, 11). Mortele et al(16) proposed that the number of lesions cannot be regarded as an indicator for distinguishing IHH from HBL because both diseases can present as solitary or multiple, and our study also supports this finding. Calcification is attributed to central hemorrhage, necrosis, or fibrosis of the lesion, especially in IHH with large size and HBL of mixed epithelial/mesenchymal type (17, 18), our study showed no statistical difference in the frequency of calcification between the two groups. As regards to the vein tumor thrombus, it may occasionally occur in HBL whereas never occurs in IHH because of their different pathological behavior, but the difference of vein involvement incidence was not significant, thus, it was a non sensitive indicator in the differential diagnosis.

The color and spectral Doppler analysis of IHH revealed a variety of flow patterns. Kassarjian et al (18) showed abnormal color flow in 60% of IHH patients and the presence of shunting was conﬁrmed in 44%. Paltiel et al (19) studied 13 children with IHH and revealed that the range of the peak Doppler shift overlapped with those previously literatures reported for malignant liver tumors. Other reported Doppler features included enlarged hepatic arteries and veins, large feeding and draining vessels surrounding or within the tumors, venous flow in some anechoic areas (18, 20). However, little attention has been paid to analysis of resistant index (RI). In our series, the color Doppler showed color flow in 8 (61.5%) IHH patients and the spectral Doppler also showed variable flow patterns: venous flow only in one case, arterial flow and/or venous flow in 7 cases, which was in accordance with published data (18-20). Although the abnormal color flow were the same commonly observed in HBL compared to IHH, the spectral Doppler showed significant difference between the two diseases, that is, the IHH was more prone to appear as arterial flow with RI < 0.7 and/or venous flow, whereas the HBL was more likely to appear as arterial flow with RI > 0.7. Histologically, the IHH is composed of numerous vessels in size from small (capillary) to large (cavernous), it contains arteriovenous malformations and venous, lymphatic or capillary components (19), thus it may act as arteriovenous shunt with relatively low RI and/or venous flow. Whereas the HBL mainly consists of numerous and disorderly hepatoblast-like cells, we speculated that lack of arteriovenous anastomosis or draining vein might be the reason for relative higher RI in HBL. Therefore, although IHH showed great variability in Doppler ultrasound, spectral Doppler might be of great utility in differential diagnosis between IHH and HBL.

Nearly 10% of HBL occurs in the neonatal period and it is also the third most common of the fetal and neonatal liver tumors (2, 21). IHH may obtain a delayed diagnosis due to absence of symptoms. Moreover, recent studies have showed IHH with elevated AFP and HBL with very low AFP level (22, 23). Thus, a combination of multiple parameters may be much more efficient. Combined the clinical features including age (< 6 months) and normal AFP level yielded high capability in differential diagnosis, with sensitivity, specificity and Youden index of 77% (10/13), 95% (36/38), and 0.72. When combined clinical features (age and AFP) and spectral Doppler as the diagnostic criterion for distinguishing these cases with positive color flow signals, the sensitivity, specificity, accuracy and Youden Index were 88%, 95%, 89% and 0.83, respectively. In other words, clinical features and spectral Doppler are useful parameters for differential diagnosis.

There were several limitations to our study. Firstly, four patients with IHH didn’t obtain pathological diagnosis owing to the risk of bleeding after biopsy and the unsuitability of surgery in patients with multiple or diffuse lesions. In addition, the pediatric liver tumors are rare diseases, so our study is a small series despite the long duration of patient collection over 10 years. Finally, none of the patients in our study underwent contrast enhanced ultrasound (CEUS). Although CEUS has higher diagnosis efficacy in focal liver lesions than baseline ultrasound, it is still a relative contraindication in children.
